# Nurses’ and clients’ perspectives after engagement in the co-designing of solutions to improve provider-client relationships in maternal and child healthcare: a human-centered design study in rural Tanzania

**DOI:** 10.1186/s12912-024-01808-0

**Published:** 2024-03-02

**Authors:** Kahabi Isangula, Eunice S. Pallangyo, Eunice Ndirangu-Mugo

**Affiliations:** 1 School of Nursing and Midwifery, The Aga Khan University, Dar Es Salaam, Tanzania; 2 School of Nursing and Midwifery, The Aga Khan University, Nairobi, Kenya

**Keywords:** User centred-design, Design thinking, User participation, Provider-patient relationships

## Abstract

**Background:**

There has been a persistent increase in clients’ dissatisfaction with providers’ competencies in maternal and child healthcare (MCH). Existing interventions have failed to address the complexity of provider-client relationships. Therefore, targeted, contextualized innovative solutions that place providers and clients at the forefront as agents of change in optimizing intervention design and implementation are needed. The study team adopted a co-design strategy as part of Human- Centered Design (HCD) approach, where MCH nurses, clients, and stakeholders partnered to design an intervention package to improve provider-client relationships in rural Tanzania.

**Objective:**

This paper explored nurses’, clients’, and MCH stakeholders’ perspectives following participation in a co-design stage of the HCD study to generate interventions to strengthen nurse-client relationships in Shinyanga Region.

**Methods:**

A qualitative descriptive design was used. Thirty semi-structured key informant interviews were conducted in the Swahili language with purposefully selected nurses, clients, and MCH stakeholders. The inclusion criterion was participation in consultative workshops to co-design an intervention package to strengthen nurse-client relationships. Data were transcribed and translated simultaneously, managed using NVivo, and analyzed thematically.

**Results:**

Three main themes were developed from the analysis, encompassing key learnings from engagement in the co-design process, the potential benefits of co-designing interventions, and co-designing as a tool for behavior change and personal commitment. The key learnings from participation in the co-design process included the acknowledgment that both nurses and clients contributed to tensions within their relationships. Additionally, it was recognized that the benefits of a good nurse-client relationship extend beyond nurses and clients to the health sector. Furthermore, it was learned that improving nurse-client relationships requires interventions targeting nurses, clients, and the health sector. Co-designing was considered beneficial as it offers a promising strategy for designing effective and impactful solutions for addressing many challenges facing the health sector beyond interpersonal relationships. This is because co-designing is regarded as innovative, simple, and friendly, bringing together parties and end-users impacted by the problem to generate feasible and acceptable interventions that contribute to enhanced satisfaction. Furthermore, co-designing was described as facilitating the co-learning of new skills and knowledge among participants. Additionally, co-designing was regarded as a tool for behavior change and personal commitment, influencing changes in participants’ own behaviors and cementing a commitment to change their practices even before the implementation of the generated solutions.

**Conclusion:**

End-users’ perspectives after engagement in the co-design process suggest it provides a novel entry point for strengthening provider-client relationships and addressing other health sector challenges. Researchers and interventionists should consider embracing co-design and the HCD approach in general to address health service delivery challenges.

**Supplementary Information:**

The online version contains supplementary material available at 10.1186/s12912-024-01808-0.

## Contribution to the field

This study pioneers an examination of the post-participation perspectives of nurses and clients (end-users) who engaged in the co-designing phase of an HCD study aimed at enhancing nurse-client relationships in rural Tanzania. Notably, there is a dearth of prior research investigating participants’ perspectives after engagement in co-designing and HCD studies in rural African contexts.

The insights derived from the perspectives of nurses and clients contribute valuable evidence to the ongoing discourse, reinforcing the argument that the co-designing process, embedded within the broader HCD framework, provides a distinctive avenue for addressing challenges within the public health paradigm and the health sector more broadly.

The perspectives of participants in this study serve as a compelling basis for advocating the widespread adoption of a co-design approach by researchers and interventionists. This underscores the critical importance of embracing this methodology to generate solutions that are not only context-specific but also highly relevant for effectively addressing challenges in health service delivery.

## Background

The healthcare system in rural African countries continue to face complex problems that negatively impact service uptake, continuity of care and adherence to medications. A major challenge is poor interactions between providers and clients in therapeutic care. Previous studies indicated there had been a persistent increase in clients’ dissatisfaction with providers’ interpersonal skills and technical competencies in maternal and child healthcare (MCH) in recent years [1–8]. Most clients’ dissatisfaction was rooted in perceived technical and behavioural incompetencies among providers [1–8]. This dissatisfaction with interpersonal and technical aspects of care continues to erode client trust in the formal healthcare system and contributes to poor healthcare service uptake, return for care, and MCH outcomes [9–10].

Various efforts have been made to address clients’ dissatisfaction using healthcare governance instruments such as policies, service charters, facility committees, complaints mechanisms, and actions of professional bodies; however, their effectiveness is not well-established [11]. As a result, punitive interventions, including employment termination, appear to remain the cornerstone of addressing this complex problem, which tends to exacerbate tensions between clients and providers [11–13]. There have also been attempts to implement competence-based interventions with a focus on improving providers’ communication skills and patient-centered care, along with patient literacy, information seeking, participation, and questioning skills. However, these efforts are often implemented on an ad hoc basis and yield unsatisfactory results [11]. The fact that existing interventions have failed to address the complexities of provider-client relationships along the MCH continuum appears to be overlooked. Other challenges that add to the complexity of nurse-client relationships include clients’ socio-economic vulnerability, poor health literacy, and behaviors; providers’ competence and behaviors; and health system challenges. This complexity necessitates novel, contextualized, and innovative solutions that place providers and clients at the forefront of intervention designs and implementation [14]. New and innovative interventions to improve the provision of high quality and satisfactory care are needed, especially in resource-constrained settings [14–16]. Ideally, to address challenges affecting the nurse-client relationship, an incremental process from intervention design to evaluation of effectiveness should be embraced. This would allow flexibility while using a standardized process that has the potential to be applied in diverse settings.

In this context, Aga Khan University invited nurses, clients, and MCH stakeholders to partner in the design of acceptable interventions to strengthen relationships using a human-centred design (HCD) approach. This is an innovative approach to problem-solving that leverages insights from the end users of new products, services, and experiences to develop best-fit solutions that are rapidly prototyped and iteratively refined [17]. There is evidence that HCD is thought to facilitate improvements in client, provider, and community satisfaction, as well as increased efficiency and collaboration in public health intervention development and implementation processes [17–19]. Furthermore, an HCD approach may result in more successful and sustainable interventions compared with traditional problem-solving approaches used in healthcare and public health [14]. The HCD approach embraces a system-wide outlook by considering interactions of factors at different levels and harmonizing individual interests to form collective interests when developing solutions. This study explored nurses’, clients’, and stakeholders’ perspectives after participation in a co-design phase to develop interventions to strengthen nurse-client relationships in MCH settings in rural Tanzania using HCD approach. The findings provide valuable evidence for researchers and interventionists, encouraging them to contemplate the utilization of co-designing and HCD approach more broadly in devising solutions for complex problems affecting the healthcare system in low-income rural contexts.

## Methods

### Design

The protocol for the parent HCD study has been previously published [20]. In summary, the parent study encompassed four key stages: (i) an exploratory community-driven inquiry using qualitative methods to unmask factors contributing to poor provider- client relationships [4]; (ii) engaging nurses, clients and MCH stakeholders in a co-design process through consultative meetings to formulate interventions targeting identified contributors; (iii) validating the emergent intervention package through qualitative methods with nurses and clients who were not engaged in previous HCD phases; (iv) iteratively refining the intervention through consultative meetings with nurses, clients and MCH stakeholders based on feedback from validation inquiry; and (v) documenting and disseminating the study’s outcomes. The current paper employs a qualitative descriptive design to scrutinize participants’ perspectives after their involvement in the co-design phase. Emphasizing the co-design process is significant, as this study marks the pioneering effort to delve into participants’ insights post-engagement in this phase, a departure from previous studies that predominantly concentrated on the outcomes of co-design initiatives (e.g. [18, 21]). We encountered a challenge in identifying a suitable theoretical framework to guide the exploratory inquiry into participants’ engagement in the co-design process. Consequently, we proceeded to examine their perspectives without relying on any specific theoretical guidance. This decision was influenced by the recognition that the HCD process inherently provides a practical investigative framework [21]. Our aim was to generate descriptive insights to address key research questions and to develop a comprehensive understanding and description of the phenomenon of engagement in co-design, without the constraints of testing an existing theory. Notably, we have previously employed this approach successfully in our research endeavors [4, 22]. Data on participants’ perspectives on the co-design process were gathered through semi-structured key informant interviews (KIIs).

### Settings

This study was conducted in Shinyanga, which is a region located in Tanzania’s Lake Zone that is predominantly inhabited by Bantus. A previous study [11] presented a detailed description of the region. Briefly, Shinyanga is a low-income region. It is administratively divided into five districts: Shinyanga Municipal Council (MC), Shinyanga District Council (DC), Kishapu DC, Kahama MC, and Kahama DC. The rationale for choosing Shinyanga for the co-design process was twofold. First, the region is predominantly inhabited by those of Sukuma ethnicity, who share a range of socio-cultural beliefs and practices with minimal diversity. Because of its near homogeneity, the region is a perfect example of many other rural regions of Tanzania. Second, despite several capacity building interventions, local data indicated there were major concerns about poor nurse-client relationships in MCH [11]. Within this region, Shinyanga MC was purposefully selected because people in these districts have access to both the formal healthcare system (mostly public and some private and faith-based facilities) and traditional care [11].

### Co-design process

During a co-designing phase of HCD study, a transdisciplinary team of purposefully selected MCH nurses and midwives, clients and MCH stakeholders including regional and municipal MCH administrators, and local non-governmental organizations the gathered for 3 days between April and May 2022 to define the challenges affecting nurse-client relationships in Shinyanga MC. The team examined the findings of a community-driven inquiry phase of HCD [4] to inform the design of an interventional package (prototype) that had the potential to improve nurse-client relationships, with consideration of acceptability and feasibility. On the first day, a *synthesis meeting* was held to review the findings of a qualitative descriptive study that examined contributors of poor nurse-client relationships [20] and share insights, experiences, and questions to generate a deeper understanding of the challenges of nurse-client relationships in Shinyanga. The second day involved an *ideation meeting* to brainstorm and generate “how might we” questions to facilitate the development of ideas for a potential solution. On the third day, a *prototype meeting* was held to evaluate the ideas generated, with consideration of pros, cons, and feasibility. This was followed by a *co-creation meeting* to develop an initial (rough) prototype model(s) as well as elements crucial to model testing (e.g., features, modality, responsible person). A total of 24 interventions were initially developed, but following a rigorous rating process, seven interventions were identified as having high potential for feasibility and acceptability. This determination was made through subjective evaluation during group discussions, taking into account the local context, as well as priorities and constraints within the healthcare system. Detailed descriptions of the rating scores can be found in a previous publication outlining the HCD outcomes [23]. In summary, the emerging interventions, along with their overall feasibility and acceptability scores, included: (i) disciplinary measures for nurses and clients (58/60); (ii) awards and recognition of nurses (56/60); (iii) re-invention of the complaints mechanism (52/60); (iv) improving the nursing curriculum (49.5/60); (v) enhancing healthcare resources (49.5/60); (vi) strengthening leadership (49/60); and (vii) improving client-centered care (48/60). Subsequently, these proposed interventions underwent the next phase of HCD, focusing on validation through insights gathered from a new group of nurses and clients [23].

### Sample size and participant recruitment

A team of 30 purposefully selected participants (10 nurses, 10 clients, and 10 MCH stakeholders) who participated in the co-design meetings were invited to KIIs after the co-design meetings. Recruitment of nurses and clients for the co-design meetings started with a courtesy visit to the Shinyanga District Medical Officer for approval to visit the purposefully selected healthcare facilities. A list of nurses, clients, and MCH stakeholders who participated in the initial study together with their phone contacts was generated. Next, the research assistants reached out to these individuals, and those who expressed interest were selected to take part in the co-design process. Although equal representation is not a primary focus in qualitative studies [24], the level and ownership of the facility (public, private, and faith-based; dispensary, health centre, and district hospital) were considered during participant enrollment for co-design meetings. Immediately after the co-design consultative meetings, participants were promptly invited to partake in KIIs as part of post-session assessment, and those expressing interest were subsequently interviewed by the research assistants and the principal investigator (PI). The inclusion criteria for this qualitative inquiry involved having actively participated in the co-design meetings. This encompassed individuals who were either a nurse working in MCH care for a minimum of two years, a client presently attending MCH clinics with a history of at least three visits within a year, or a regional or municipal MCH service administrator or a representative of non-government organizations dealing with MCH in Shinyanga. It is noteworthy that all participants willingly agreed to participate in the study, and there were no refusals. This positive response may be attributed to their enthusiasm to share perspectives, particularly given that this was their first experience engaging in a study using HCD approach and the flexibility of conducting interviews within a week after co-design meetings.

### Data collection tool

A semi-structured guide for the KIIs was developed and translated through a consultative process involving experts at Aga Khan University. The English version of the interview guide was translated into the Swahili language then back translated to English and checked for conceptual equivalence. The guiding research question was: “How did you perceive your participation in the co-design process?” Questions in the interview guide ranged from participants’ insights on the co-design process including its potential benefits, key learnings from the process, how their engagement in co-design have shaped their behaviors and decisions, how they would use the skills gained through participation in co-design, and any recommendations. The interview guide underwent pre-testing in two healthcare facilities within the study settings, after which nurses and clients from these institutions were subsequently excluded from participating in the HCD study. After pre-testing, the guide was refined to ensure readiness for use in the actual data collection process.

The research team comprised three members: the Principal Investigator (PI), a distinguished medical doctor and public health expert serving as an assistant professor with expertise in qualitative research and interpersonal relationships, along with two senior nurses/midwives who are associate professors with esteemed track records in nursing practice and MCH care within low-income countries. Additionally, three research assistants with Diplomas in health sciences were recruited and trained on the HCD process and techniques about this study and were used to conduct KIIs for this study alongside the PI. Close and supportive supervision of the research assistants was implemented by the PI throughout data collection and analysis to ensure data quality.

### Data collection

The semi-structured KIIs took place during the first week following the co-design meetings, arranged at locations and times preferred by the participants. For nurses and clients, interviews were conducted in quiet and private rooms within the healthcare facility where they worked or received care. MCH stakeholders’ interviews were conducted in their respective offices. Before the KIIs, participants were given information about this study and the risks and benefits of participation (an information sheet was part of the interview). Verbal consent for the interview and voice recording was sought in advance and recorded as part of the interview. Next, an interview session lasting approximately 30–40 min was conducted. It is crucial to note that our sample size was adequate to achieve data saturation. Additionally, the research team also maintained field notes, which aided in developing the methodology section of this paper with careful consideration of what transpires data collection. Because of COVID-19, all participants and research assistants were provided with face masks and hand sanitizers, and social distancing was maintained throughout the interviews.

### Data management and analysis

Data transcription and translation occurred simultaneously by the research assistants. After translation, the PI verified the translation and generated pseudonyms for each transcript. Data analysis utilized the deductive thematic analysis method, following the framework outlined by Braun and Clarke [25]. The choice of a deductive thematic analysis approach was driven by the existence of a clear research question and the objective to explore participants’ favorable views towards HCD in this rural context. Additionally, time constraints, as the study approached the conclusion of the funding period, played a role in this decision. In the deductive thematic coding process, the research team initially examined the research questions and collaboratively generated several initial themes based on consensus. This iterative process resulted in the development of an analytical matrix encompassing the main themes (key lessons, benefits of co-design, and its influence on behavior and personal commitment) and their corresponding subthemes. Subsequently, the PI reviewed individual transcripts and identified phrases (codes) that encapsulated participants’ responses to investigators’ probes. These codes were then exported to the relevant themes and related subthemes using NVivo software (QSR International Version 12). Peer engagement was sustained throughout the coding process, employing a consensus-based approach within the research team to decide whether to include codes that did not align with the developed subthemes and themes or to discard those subjectively and objectively deemed of no critical value to this study. Finally, the coded data were exported to MS Word for the generation of the research report.

## Results

### Participants’ demographics

A detailed description of participants’ demographics is presented in Table 1 of our recent publication [23]. Briefly, consultative meetings for co-designing phase involved 30 participants (90% female), with most participants aged 21–30 years. Most participants were seeking or providing care at a health centre (40%). On the one hand, most nurses and MCH stakeholders had a higher level of education level (nurses and stakeholders with college and above were 90% of co-design participants) as compared to clients (clients with secondary and below were 70% of co-design participants). To mitigate educational-based power dynamics, we established a requirement for a client to serve as the chairperson of the group. The chairperson was responsible for moderating the group discussion and presenting findings to the larger groups. Additionally, a research assistant was assigned to each group to facilitate and ensure active participation of clients (Table 1).

**Table 1:** Participants’ demographics (*N* = 30)

		N	%
Participant category	Nurses Clients Stakeholders	10 10 10	33 33 33
Gender	Female Male	27 3	90 10
Age, years	21–30 31–40 41–50 > 50	18 9 2 1	60 30 7 3
Level of the facility of practice/care seeking	Hospital Health centre Dispensary	8 12 10	27 40 33
Ownership of facility of practice/care seeking	Public Private Faith-based	17 8 5	57 27 16
Level of education	Nurses	Clients	Stake holders
None	0	1	0
Primary	0	3	0
Secondary	1	3	1
College	6	2	4
University	3	1	5

### Summary of key themes

The analysis revealed three main themes. Table 2 presents a concise overview of each theme along with their related subtheme and each of these themes are examined in detail below.

**Table 2:** Key themes and sub themes

Theme	Subtheme(s)/descriptions
Key learnings from the co-design process	• Both nurses and clients as contributors to sour therapeutic relationship • Benefits of good therapeutic relationships extend to the health sector • Interventions for improving nurse-client relationship need to focus to nurses, clients, and health sector
Benefits of co-design approach	• Innovative way of jointly designing solutions • Facilitate designing of effective and impactful solutions • Simple, effective, friendly, and feasible • Offers opportunity for co-learning among parties
Co-designing as a tool for behaviour change and personal commitment	• Create awareness of individual weakness and areas for improvement • Shape behaviors before actual implementation of solutions • Shape personal commitment to make changes in their practices

### Theme 1: key learnings from participation in the co-design process

Although the focus of the consultative meetings was on generating an intervention to strengthen nurse-client relationships, participants offered wealthy descriptions indicating that they benefited by learning through engagement in the co-design process. Looking across transcripts for the descriptions of key learnings after participating in the co-design process, they reveal similarities across participant groups (nurses, clients, and MCH stakeholders). The focus on this theme predominantly revolves around the broader HCD study topic—*provider-client relationships*—rather than the intricacies of co-designing itself. Key learnings related to the broader HCD study focus were threefold. The first key learning was the acknowledgment that both nurses and clients contributed to tensions in their therapeutic relationships. For example, a nurse in a primary healthcare facility became “aware of the challenges that make providers not able to provide friendly services to their clients” with an acknowledgement that “these challenges arise from either the nurse, client, or inadequate resources.” Notably, participants’ descriptions cemented the need for bringing together both parties when seeking to develop impactful solutions. The second key learning was a broad cognition that the benefits of good nurse-client relationships citing improved trust, friendship, and shared decision-making as examples, extended beyond nurses and clients to the health sector. Nearly all participants concurred that healthcare facilities and the health sector derive equal or even greater benefits from improved nurse-client relationships compared to the benefits experienced by nurses and clients themselves. The third key learning was a recognition that the improvement of nurse-client relationships required interventions that target nurses, clients, and the health sector. This partly explains why the emerging intervention during co-designing encompasses those focusing on nurses, clients, and health sector. There was broad consensus among participants that interventions that target nurses could include awards and recognition to build their morale, improvement and timely payment of salaries and allowances and improvement of the nursing curriculum. Similarly, interventions focusing on clients included education on their rights and client-centred care. In addition, interventions focusing on the health sector included strengthening complaints mechanisms, increasing human resources for health, and ensuring the availability of medical supplies. Some of the key learnings described by participants are seen in the following quotes:

*“I learned that as a client, I have my rights and that having a good relationship with my nurse can create trust and friendship that can facilitate nurses to give me appropriate care and build nurses’ confidence and morale.” (Client, Dispensary)*.

*“I learned that improving nurse-client relationship needs addressing the challenges faced by nurses such as delayed payment…but also awarding those who perform well. I also learnt that we need to educate clients on their rights and provide client-centred care. We also need to ensure the availability of an adequate number of nurses and medicines.” (MCH Leader)*.

### Theme 2: the benefits of co-design approach

A notable finding from participants’ descriptions highlights the advantages of employing co-design as an approach for devising solutions to tackle healthcare challenges. There was a broad consensus among participants that co-designing is very beneficial and, is a promising strategy for addressing many challenges facing the health sector beyond interpersonal relationships. The justifications for considering co-designing as beneficial and promising were fourfold. First, co-design was viewed as an innovative approach that offered an opportunity for parties impacted by the problem to meet and jointly generate interventions that are acceptable for all. Second, there was a unanimous agreement that co-designing enables the exploration of individual experiences, perspectives, and insights of the parties involved in a problem. This, in turn, facilitates not only the development of effective and impactful solutions but also improving the relationships among parties involved as they interact to design solutions. Relatedly, co-designing was regarded as facilitating peace of mind among participants because they are fully engaged in developing solutions. A client and a nurse commented:

*“This is a very innovative approach, and it is an effective approach for addressing the challenges in the health sector because people from either side meet, discuss and agree on the solution. The participants learnt that in the process, their relationship improves as they meet.” (Client, Hospital)*.

*“This is a very good approach because it brings insights of the people who are affected by the problem. For example, nurses and clients can sit and develop solutions that can bring positive change. This is because, the solutions they develop touch everyone directly, which is very important for successful implementation.” (Nurse, Dispensary)*.

Third, co-designing was regarded as being highly likely to result in feasible and acceptable solutions for addressing healthcare challenges beyond the therapeutic relationship. Almost all nurses, clients, and MCH stakeholders considered co-designing as “simple,” “effective,” “friendly,” and resulting in “feasible” solutions. Nurses suggested that co-design facilitated consensus building, satisfaction, and acceptance of the emerging solutions, which is critical for successful implementation. Fourth and final, co-designing was considered to facilitate gaining new insights, knowledge, and skills during their engagement in the co-design process. In other words, co-designing was considered to offer a co-learning opportunity for participants as they interacted to analyze problems and generate solutions. Consequently, more than half of the participants suggested that “other healthcare partners needed to embrace co-design in addressing healthcare challenges” instead of coming up with prescribed solutions. One MCH stakeholder commented:

*“Co-designing is very innovative because everyone is fully engaged in giving insights to improve health services. They build a consensus together, for example, nurses, clients, and leaders are fully engaged…other partners need to consider this approach instead of coming up with pre-determined solutions that may not be successful.” (MCH Leader)*.

### Theme 3: co-design as a tool for behavior change and personal commitment

A novel finding from this study is the participants’ descriptions that underscore co-design as an influential tool for advocating behavioral changes and fostering personal commitments. Many participants shared narratives about how their involvement in co-designing sparked a transformation in their own behaviors. They went ahead to describe a range of commitments to become agents of good practices from this point forward by applying the key learnings gleaned from their participation in the co-design process. For instance, the majority of nurses expressed that co-designing played a crucial role in unveiling and increasing their awareness of their weaknesses, encompassing both behavioral and attitudinal aspects, and identifying areas for improvement. As a result, nearly all nurses affirmed a commitment to transforming their behaviors towards clients. This commitment entailed a dedicated effort to cultivate positive relationships with their clients, involving tangible changes in their behaviors and practices within healthcare settings. More specifically, most nurses committed themselves to providing better health services, improving efficiency, upholding clients’ rights, avoiding client discrimination, adhering to nursing ethics, and increasing closeness with their clients. Furthermore, nurses were committed to taking a sensitization role by educating peers who did not take part in the co-design process on the benefits and strategies for developing good nurse-client relationships, as well as educating clients on how to improve therapeutic relationships. A nurse working in a dispensary (a lowest primary healthcare facility) commented:

*“Participation helped me to know the things I have been doing wrong. I will strive to offer quality care and adhere to nursing ethics…I will offer care without discrimination and ensure that I respect and uphold the rights of my clients…I will also encourage my peers to adhere to nursing ethics and fulfil their responsibilities effectively so that we can have a good relationship with our clients.” (Nurse, Dispensary)*.

Moreover, clients detailed shifts in their behaviors concerning interactions with nurses, pledging to become proactive agents of change. This commitment manifested in their promise to arriving promptly at the MCH clinic, adhering strictly to established service delivery procedures, articulating their medical concerns more clearly, and fostering mutual respect and closeness with the nursing staff. Clients also committed to taking a sensitization role by giving feedback about the process to community leaders and educating their peers who did not take part in the co-design process on how to minimize tensions with nurses. One client receiving MCH care at a health center commented:

*I changed a lot because of participation. From now, I will start arriving at the facility early, use friendly language towards nurses and offer clear information…I will use the skills I gained to educate my fellow clients and surrounding community on how to build good relationships with our providers…” (Client, Health Center)*.

Additionally, MCH stakeholders expressed a transformation in their behaviors regarding interactions with their direct supervisees. These stakeholders pledged to amend their leadership practices by ensuring the effective fulfillment of their duties, upholding principles of equity and staff rights, following established guidelines, and employing discussions and consensus-building to address conflicts involving nurses and clients. Most went ahead to express a commitment to taking a sensitization role by educating providers on how to strengthen their relationship with clients, using meetings to influence the implementation of solutions generated, conducting mentorship to providers on good customer care, and continued monitoring of clients’ complaints. One MCH stakeholder commented:

*“The knowledge and skills I gained have helped me a lot already. As a leader, I will use the knowledge gained to fulfil my leadership role equitably…not discriminating my staff…offer mentorship to my staff and closely monitor complaints so that we can have good customer care to our clients.” (MCH Leader)*.

Collectively, these narratives underscored the significant knowledge and skills acquired by participants during their involvement in the co-design process. Participants recognized co-design as a potentially impactful strategy for tackling healthcare challenges extending beyond nurse-client relationships. The belief was that involving all relevant parties in the problem-solving process would ensure full commitment to the solution’s development and utilization. Co-design was further seen as a catalyst for behavioral change, fostering personal commitment to enhancing individual practices and assuming advocacy roles by educating peers who were not part of this process. As a result, the suggestion was put forth for other implementation partners to consider integrating co-design into their approaches for addressing challenges in health service delivery.

## Discussion

The aim of this study was to explore participants’ viewpoints following their participation in the co-design phase of a HCD study, designed to devise solutions for enhancing provider-client relationships in the context of MCH care in rural Tanzania [4, 23]. In reflecting on their experiences, participants highlighted significant insights gained from their involvement in the co-design process, particularly concerning patient-provider relationships. They underscored the potential advantages of co-design in addressing tangible issues and portrayed engagement in co-design as a catalyst for participants to modify their behaviors and reinforce their commitment to enhancing practices, even before the practical implementation of the emerging solutions. This implies that the act of co-designing has a direct impact on the behaviors and practices of participants. Moreover, considering that the parent study pioneered in embracing the HCD approach to fortify provider-client relationships [4, 23], delving into the perspectives of nurses and client’s post-engagement in co-designing is a vital stride in accumulating evidence for its broader application in tackling healthcare challenges that necessitate the involvement of end-users in the devised solutions.

As previously mentioned, the results revealed that engagement in the co-design process promoted learning among nurses, clients, and MCH stakeholders. Participants gained awareness that challenges in therapeutic relationships originated from both providers and clients. Moreover, they recognized that the benefits of positive provider-client relationships extend to the overall health sector. Another insight articulated by co-design participants is that addressing tensions in therapeutic relationships requires involving the experiences, insights, and perspectives of providers, clients, and key health sector stakeholders through collaborative efforts to develop acceptable solutions. These findings align with earlier studies whose primary focus were to document the contributions of better therapeutic relationships on providers, clients and health system [11, 24, 26–27]. For instance, a prior study in a comparable setting revealed that inadequate provider-client relationships result from contributions by both providers and clients, as well as challenges within the healthcare sector [11]. This study also emphasized that a positive and trusting relationship between providers and clients yields various benefits. Clients experience improvements in healthcare-seeking behaviors, disclosure, adherence, and continuity of care. Providers benefit from enhanced confidence, work morale, and reputation. Furthermore, the healthcare sector, particularly healthcare facilities, sees increased income and societal reputation [11]. The depiction of key lessons learned from the engagement in the co-designing process places co-learning at the core of the participants’ learning experience. This suggests that participants in co-designing not only contribute their insights and experiences, fostering the development of effective solutions, but also acquire a broader understanding of the problems and recognize their own contributions to its persistence. This fundamental aspect of co-designing within the context of HCD has been previously emphasized [17–19, 28–21, 29], partially explaining the uniqueness of this innovative approach to addressing healthcare challenges.

There seems to be a restricted emphasis on understanding the perspectives of participants in the co-design and HCD processes. Many previous studies tend to concentrate more on documenting the iterative processes of HCD and co-designing and their outcomes (prototypes or interventions) without delving into how participants perceive these approaches [28, 30–31].The findings of this study bridges this gap by indicating that co-design was widely acknowledged as a promising approach for addressing many challenges facing the health sector beyond interpersonal relationships. This was because co-design was considered an innovative approach and also offered an opportunity for parties impacted by the problem to meet and generate acceptable interventions for all concerned. Co-design was considered to facilitate “peace of mind” among participants because they were fully engaged in analyzing the problem and generating solutions that considered individual experiences, perspectives, and insights. Consequently, co-design was regarded as a simple, friendly, and effective way to develop feasible and acceptable solutions and offers opportunity for co-learning among parties involved in a problem. These findings resonated with previous scholarly insights about co-design processes. As a central aspect of HCD, co-design is considered to facilitate improvements in client, provider, and community satisfaction, as well as increase efficiency and collaboration in public health intervention development and implementation processes [17–19, 32–33]. The approach takes a system-wide outlook by considering interactions of factors at different levels and harmonizing individual interests to form collective interests when developing solutions. Intrinsic to the co-design process is the fact that end-users jointly understand a problem, act on it, and learn from working collaboratively to contest power relationships and effect change. The co-design approach is a highly adaptive and creative approach to problem-solving and enables the team to understand the problem and ensure that all relevant stakeholders are at the forefront as solution designers [33] more deeply. Consequently, the emerging solution package (prototype) can be more successful and sustainable compared with traditional problem-solving approaches in healthcare and public health [17]. These findings solidify the existing compelling evidence, advocating for the widespread adoption of a co-design approach by researchers and interventionists.

A crucial aspect of co-design is its potential as an effective tool for behavior change and personal commitment among participants [33]. This reinforces the idea that co-designing can foster learning among participants, subsequently influencing their behaviors and practices even before the implementation of the emerging solution. This strength of co-designing emerged strongly in this study, as almost all participants affirmed that co-designing facilitated acknowledgement of “things they have been doing wrong” and they had gained new knowledge and skills as they interacted to analyze problems and generate solutions. As a result, all participants affirmed changing their behaviors and committed to become agents of change by changing their practices. For example, nurses described becoming more aware of their weaknesses, affirmed changing how the treat clients from this point and committed to developing good relationships with their clients by providing better health services, improving efficiency, upholding clients’ rights, avoiding client discrimination, adhering to nursing ethics, and increasing closeness with their clients. Nurses also committed to sensitizing and educating their peers and clients on the benefits of and strategies for improving therapeutic relationships. Clients affirmed changing their behaviors that contributed to tensions in their relationships and committed toeducating fellow clients on how to improve their relationships with their nurses. Similarly, MCH leaders committed to changing their leadership practices to more favorable and non-discriminatory practices as well as providing mentorship for providers and closely monitoring client complaints. As a result of this acknowledgement, participants overwhelmingly recommended the adoption of the co-design approach among other implementing partners in addressing challenges in the health sector instead of adopting pre-developed solutions that may not be successful. Together, these findings suggest that involvement in co-design could serve as a crucial catalyst for behavior and practice changes among participants, turning them into advocates for the implementation of the emerging solutions.

## Limitations

This study had some limitations. The co-design approach used nurses as an exemplar of providers to partner with clients and MCH stakeholders to co-develop a prototype for strengthening interpersonal relationships in MCH in a rural setting. However, patients interact with a multidisciplinary team of providers in healthcare settings. Conducting a similar study with clients and stakeholders partnering with other providers (e.g., doctors, pharmacists, lab personnels etc.) and in a different setting may yield different experiences and insights. However, as this is the first such study in this context, further inquiries may extend beyond the nursing profession and rural contexts. Furthermore, the co-design approach appeared to be a new concept for many healthcare sector stakeholders. Therefore, capacity building on the co-design steps may be needed for researchers and health sector actors before it can be fully employed as a tool for generating solutions for complex challenges in the healthcare system. Relatedly, our desire to capture the insights of individuals involved in the co-design process led us to interview the same participants engaged in the co-design activities. While this could potentially introduce selection bias, we consider it unavoidable due to the specific focus of our study and the limited application of the co-design approach in a similar topic and context, consequently limiting the pool of potential participants. As co-design gains recognition in comparable contexts and topics, future studies may explore triangulating findings by involving individuals who have previously engaged in co-design, extending beyond the specific focus on therapeutic relationships. Finally, bringing together end-users of the solution or people directly impacted by the challenge may have significant financial implications. However, there is an increasing number of international organizations that are willing to fund interventions embracing co-design approaches for generating solutions to address healthcare challenges.

## Conclusions

In conclusion, end-users’ perspectives after engagement in the co-design process suggest that it provides a novel entry point for strengthening provider-client relationships and addressing other health sector challenges, as clients are invited to partner with providers and stakeholders in the design of highly acceptable and feasible interventions. The co-design process provides a co-learning opportunity which facilitates understanding of areas that need improvement, and influences change in behaviours and practices among participants, thereby making them agents of change before an emerging solution has been implemented. Therefore, researchers and interventionists need to embrace the co-design strategy and HCD approach more broadly in addressing health service delivery challenges.

### Electronic supplementary material

Below is the link to the electronic supplementary material.

**Supplementary Material 1:** Data Collection Tool

## Data Availability

The dataset(s) supporting the conclusions of this article are) included within the article (and its additional file(s)). Additional data on the HCD process that are not part of the published article will be available on request from the AKU through the corresponding author (KI). Some data may not be publicly available for ethical reasons (i.e., information that could compromise the privacy of research participants).

## Abbreviations

HCD: Human-centered design
MCH: Maternal and child health
KII: Key informant interview
