# Supplementary material for: Nurses’ and clients’ perspectives after engagement in the co-designing of solutions to improve provider-client relationships in maternal and child healthcare: a human-centered design study in rural Tanzania
Source: BMC Nurs. 2024 Mar 2;23:148. doi: 10.1186/s12912-024-01808-0 (PMC10908081; doi:10.1186/s12912-024-01808-0)
Supplement: Supplementary file 1 — Supplementary Material 1: Data Collection Tool [file 12912_2024_1808_MOESM1_ESM.docx]

**DATA COLLECTION TOOL**

| **PROJECT TITLE:** **IMPROVING NURSE- CLIENT RELATIONSHIPS IN MATERNAL AND CHILD HEALTH CARE IN RURAL TANZANIA: A HUMAN CENTERED DESIGN (HCD) APPROACH** |
| --- |

**DEMOGRAPHIC INFORMATION**

| AGE | GENDER | MARITAL STATUS | LEVEL OF LICENCE  (NURSES ONLY) | HIGHEST LEVEL OF EDUCATION | LEVEL OF HEALTH FACILITY | YEARS OF MCH CARE EXPERIENCE |
| --- | --- | --- | --- | --- | --- | --- |
|  |  |  |  |  |  |  |

**KII QUESTIONS**

1. How did you participate in co -designing?

(SWAHILI: Ulishiriki vipi kative ubunifu shirikishi?)

1. How did you perceive your participation in the co-design process?

(SWAHILI: Una fikra zipi kuhusu njia hii ya ubunifu shirikishi?)

1. How have you benefited from participation in the co-design meetings? What have you learned from taking part in the process?

*(SWAHILI:Umenufaikaje/vitu gani umejifunza kutokana na ushiriki wako katika vikao vya ubunifu shirikishi?)*

1. How will you use what you learnt from participation in the co-design process in improving the relationship with your nurse/client?

*(SWAHILI: Utatumiaje maarifa uliyojifunza katika kuimarisha mahusiano na wateja au wauguzi wako?)*

1. What are your suggestions about the use of co-design process (human centred design) in designing solutions for challenges facing the health sector?

*(SWAHILI: Maoni kuhusu utumiaji wa ubunifu shirikishi (Human Center Design) katika kutengeneza mikakati ya kutatua changamoto katika sekta ya Afya?)*

1. What are your suggestions about the whole research process?

*(SWAHILI: Maoni kuhusu mchakato mzima wa utafiti?)*

THANK YOU End Time_________________________
